# An AI-powered framework for assessing teacher performance in classroom interactions: a deep learning approach

**DOI:** 10.3389/frai.2025.1553051

**Published:** 2025-09-03

**Authors:** Arwa Almubarak, Wadee Alhalabi, Ibrahim Albidewi, Eaman Alharbi

**Affiliations:** ^1^Department of Computer Sciences, Faculty of Computing and Information Technology, King Abdulaziz University, Jeddah, Saudi Arabia; ^2^Ministry of Education, Riyadh, Saudi Arabia; ^3^Center of Research Excellence in Artificial Intelligence and Data Science, King Abdulaziz University, Jeddah, Saudi Arabia; ^4^Immersive Virtual Reality Research Group, King Abdulaziz University, Jeddah, Saudi Arabia

**Keywords:** education, computer vision, deep learning, object detection, in-classroom interaction, improving classroom teaching, teacher performance evaluation, teacher performance

## Abstract

**Introduction:**

Teacher performance evaluation is essential for improving instructional quality and guiding professional development, yet traditional observation-based methods can be subjective, labor-intensive, and inconsistently reliable. This study proposes an AI-powered framework to objectively assess classroom interactions.

**Methods:**

We developed and evaluated a computer-vision framework using three state-of-the-art object detectors—YOLOv8, Faster R-CNN, and RetinaNet—to identify eleven classroom interaction categories. A labeled dataset of 7,259 images collected from real classroom settings was annotated and used for training and evaluation. Performance was assessed using mean Average Precision (mAP).

**Results:**

YOLOv8 achieved the best performance among the evaluated models, with an mAP of 85.8%, indicating strong accuracy in detecting diverse classroom interactions. Faster R-CNN and RetinaNet performed competitively but were outperformed by YOLOv8.

**Discussion/Conclusion:**

The results demonstrate that modern deep learning–based detection can provide more objective and reliable insights into teacher–student interactions than traditional approaches. The proposed framework supports evidence-based evaluation and has the potential to enhance feedback and outcomes in educational practice.].

## 1 Introduction

In the educational process, teacher performance evaluation is crucial for improving instructional quality, ensuring accountability, and supporting professional development. Traditional methods, such as classroom observations and test-based metrics, are limited by subjectivity, evaluator bias, and insufficient feedback (Fernández and Martinez, 2022). These conventional approaches often fail to capture the full complexity of teaching practices and student needs, making them inconsistent and lacking in actionable insight for teachers. As a result, they hinder opportunities for continuous improvement and professional growth.

Teacher evaluations also contribute to educational equity by holding all educators to consistent standards, which is especially important in addressing achievement gaps among diverse student populations. Through targeted professional development, instructors can improve their instructional strategies and enhance student outcomes (Fan, 2024). A robust evaluation system further supports accountability within educational institutions by informing personnel decisions such as promotion and tenure. By integrating both formative and summative assessment methods, such systems provide a more comprehensive evaluation of teaching effectiveness, fostering an environment of continuous growth and excellence (Close et al., 2020).

During the evaluation process, it is essential to consider the teacher’s Key Performance Indicators (T-KPIs) according to the adopted educational system. T-KPIs play a vital role in assessing educators, ensuring that students receive a high-quality education and contributing to overall improvements in educational standards (Kardianto et al., 2022; Taylor and Tyler, 2012; de Almeida, 2017; Pelayo et al., 2022). Several studies have examined the application of T-KPIs in various countries, including Saudi Arabia (Hakim, 2015), Indonesia (Setyaningsih and Suchyadi, 2021), Australia (Stacey et al., 2020), China (Ding et al., 2021), India (Patel, 2018), and others (Guo et al., 2021). T-KPI forms provide evaluators with immediate and specific feedback, helping to identify both teachers’ strengths and areas for improvement. However, these forms alone often fail to deliver timely, objective, and comprehensive insights into classroom dynamics. Nevertheless, when used alongside other evaluation methods, T-KPIs contribute to a more holistic understanding of teacher performance (Amzat, 2017).

Traditional methods for evaluating teacher performance—such as classroom observations, test-based metrics, and manual KPI forms—have notable limitations. These include subjectivity, evaluator bias, limited feedback, and the inability to capture real-time or nuanced classroom dynamics. Evaluations often rely on standardized test scores, which fail to reflect the complexity of teaching practices and students’ diverse needs. Additionally, such methods are typically time-consuming, inconsistent, and offer limited actionable insight for teachers, thereby hindering professional growth and continuous improvement.

In the literature, teacher evaluation in the classroom revolves around three phases: observation of the teaching process using T-KPI forms (cards), manual analysis of the video recordings, and automated and intelligent analysis of the videos using Artificial Intelligence (AI) (Williams and Hebert, 2020). Observations in the classroom provide immediate, contextual feedback and insight into classroom dynamics. Nevertheless, these methods may be time-consuming, introduce observer bias, and be challenging to scale up (Gitomer et al., 2014). Manual video analysis has been explored as a richer alternative, yet it too is constrained by time demands, the need for trained observers, and privacy concerns. These constraints make it difficult to scale evaluations reliably or provide timely feedback. As a result, there is growing interest in leveraging AI-driven approaches to overcome these challenges through objective, scalable, and data-rich evaluations (Heard and Peltier, 2021).

Traditional methods for evaluating teacher performance have significant shortcomings that undermine their effectiveness and fairness. According to Steinberg and Kraft (2017), these conventional evaluation methods rely heavily on subjective observations and infrequent assessments, leading to inconsistent and unreliable evaluations that may not accurately reflect a teacher’s day-to-day performance or growth. Consequently, teachers may experience significant stress that led to performance anxiety and may further distort the evaluation results. Similarly, Wei et al. (2023) emphasizes that traditional methods are commonly based on standardized test scores, which fail to capture both the multifaceted nature of teaching and the diverse needs of students. These methods frequently ignore important aspects of teaching, such as the development of critical thinking and socio-emotional skills, which standardized tests cannot measure. Furthermore, traditional evaluations typically offer limited feedback, providing little actionable insight for teachers to improve their practice. This lack of constructive feedback impedes professional and continuous development. Moreover, traditional teacher evaluations rarely offer actionable insights for teachers. Overall, these studies highlight the need for holistic and continuous evaluation approaches that accurately improve teachers’ professional development and reflect their contribution to student learning.

The emergence of AI technology presents a transformative approach to evaluating educational performance, offering new opportunities to improve teacher evaluation. AI enables the analysis of large volumes of data from various sources—such as classroom interactions, student performance metrics, and instructional materials—facilitating a more comprehensive understanding of teaching effectiveness. By identifying patterns in teaching methods and engagement levels, AI algorithms can more accurately and objectively assess educators’ strengths and areas for growth. Moreover, AI-driven evaluations offer the advantage of immediate feedback, allowing teachers to adjust their practices to better meet students’ needs (Owoc et al., 2021). Ongoing research suggests that integrating AI into teacher evaluations can enhance both their quality and fairness, positioning this integration as a promising research area (Ding et al., 2024). However, concerns such as privacy, algorithmic bias, and the interpretability of AI-generated assessments must be addressed. To overcome the limitations of traditional evaluation methods, researchers have introduced increasingly accurate, data-driven, and scalable AI techniques. Steinberg and Kraft highlight the use of AI in analyzing classroom interactions, offering real-time feedback and pinpointing areas for improvement (Steinberg and Kraft, 2017). Heard et al. explore video-based evaluations that support teacher self-reflection and peer review, improving the reliability of observations (Heard and Peltier, 2021). Similarly, Kane et al. (2020) emphasizes the value of continuous data analytics in tracking student progress and evaluating teacher effectiveness in a dynamic and responsive manner. These technologies help reduce biases inherent in manual evaluations and generate actionable insights for personalized professional development. Collectively, these advances reflect a shift toward a more supportive and nuanced evaluation framework aligned with modern educational goals. In addition to automating routine tasks such as grading, AI systems can provide real-time feedback, further enhancing teaching effectiveness and productivity (Guo et al., 2021; Lee et al., 2024).

T-KPI analysis is essential for enhancing educational outcomes, ensuring accountability, supporting professional development, and enabling evidence-based decision-making (Guo et al., 2021; Ali, 2024). Five commonly used T-KPIs include: (a) student achievement, (b) instructional quality, (c) professional development and growth, (d) collaboration and communication, and (e) classroom environment. AI-based systems offer objective and reliable evaluations of these indicators, helping identify areas for improvement and raising the overall quality of education (Guo et al., 2021).

Several AI technologies are currently being explored to support teacher performance evaluation through comprehensive and data-driven insights. These technologies are typically classified by domains and methods. Domains such as Computer Vision (CV), image processing, and the Internet of Things (IoT) enable the collection and analysis of visual and sensory data, allowing real-time monitoring of classroom interactions. Meanwhile, methods like Machine Learning (ML) and Deep Learning (DL) play a pivotal role in interpreting this data, enabling pattern recognition, predictive modeling, and automated evaluations. When applied effectively, these approaches provide deeper insights into teaching practices, student engagement, and classroom dynamics, contributing to more informed decisions and improved educational outcomes (Guo et al., 2021; Ahmed et al., 2022; Tuli et al., 2019).

Computer Vision (CV) offers a valuable means of assessing teacher performance by tracking movements, gestures, facial expressions, and interactions within the classroom. Through object detection and video analysis, it is possible to monitor behaviors such as teacher positioning, student engagement, use of visual aids, and overall participation patterns—providing objective insights into teaching style and classroom dynamics. This automated approach enables consistent, scalable analysis, supporting more comprehensive and timely teacher evaluations (Vijayakumar and Vairavasundaram, 2024).

Beyond computer vision (CV), emerging technologies such as the Internet of Things (IoT), Machine Learning (ML), and Deep Learning (DL) significantly expand the scope of teacher performance evaluation. IoT devices—such as smart cameras, microphones, and wearable sensors—can provide contextual data by monitoring environmental factors like classroom noise levels and student movement. ML models help uncover patterns and correlations in classroom behavior, while DL techniques can extract subtle cues from video and audio recordings, such as detecting emotional tone or identifying specific teaching strategies. Together, these technologies enable more nuanced, real-time, and scalable evaluations, surpassing the limitations of traditional observational methods (Kaur and Singh, 2023). However, traditional approaches to monitoring in-classroom interactions remain limited in their ability to track critical metrics, such as textbook usage, types of student participation, and teacher-student dynamics. As a result, teachers may struggle to manage classroom interactions effectively, leading to suboptimal instructional outcomes.

The primary objective of this study is to automate the evaluation of teacher performance by detecting in-classroom interactions using AI-driven models. This initiative builds upon previous research (Almubarak et al., 2024), which proposed the foundations of a comprehensive teacher evaluation framework. By constructing a labeled dataset of classroom interactions and comparing multiple object detection models, this work aims to demonstrate the feasibility and effectiveness of automated, data-driven performance assessments.

This study introduces a computer vision and deep learning-based evaluation system using object detection algorithms (YOLO, Faster R-CNN, RetinaNet). It addresses the following research questions:

*RQ1.* Can the proposed model detect student participation reliably?

*RQ2.* Can the proposed model identify textbook usage accurately?

*RQ3.* Can the proposed model track student and teacher activity effectively?

Regarding the research hypothesis, we predicted that the proposed model could accurately and reliably detect classroom interactions and evaluate teacher performance regarding student engagement and activities. The research questions will be answered during the following sections, and the hypothesis will be tested.

While this study addresses the long-standing limitations of traditional evaluation practices, the technical limitations of the proposed AI-based detection model identified through experimentation—are discussed in detail in the Discussion section.

This study makes several key contributions to the field of AI-driven teacher performance evaluation. First, a labeled image dataset has been created to capture various teacher and student interactions and behaviors within a classroom environment. The dataset includes detailed annotations for activities such as **Closed-Book, Electronic-Book, No-Book, Opened-Book, Raising-Hand, Student-Answers, Student-Reads, Student-Writes, Teacher-Explains, Teacher-Follows-up-Students, and Worksheet**, providing a comprehensive representation of classroom dynamics. Second, the performance of this dataset has been evaluated using multiple object detection algorithms, enabling a comparative assessment of their accuracy, robustness, and suitability for recognizing these specific behaviors and interactions.

While the object detection networks employed (YOLOv8, Faster R-CNN, RetinaNet) are well-established, the novelty of this study lies in their adaptation to the educational domain—specifically for detecting pedagogically meaningful interactions aligned with teacher performance indicators (T-KPIs) in real classroom settings.

Lastly, the study explores the practical applications of the system in real-world classroom settings, discussing its potential benefits for automated teacher assessments, identifying classroom engagement patterns, and supporting data-driven educational improvements. Additionally, the study highlights existing limitations and provides recommendations for future enhancements based on experimental findings.

The major contributions of this study are:

- The development of a labeled image dataset capturing various teacher-student interactions.
- The evaluation of the dataset using various object detection algorithms to assess their performance in recognizing these behaviors.
- The discussion of system applications, limitations, and recommendations for future enhancements.
- The adaptation of general-purpose object detection networks to the educational domain, using expert-driven labels and real classroom footage to detect pedagogically meaningful interactions—establishing a foundation for automated teacher performance scoring.

The rest of this article is organized as follows. Section 2 reviews the related works. Section 3 introduces the research materials and methods. The results, discussion, and conclusion are illustrated in Sections 4, 5, and 6, respectively.

## 2 Literature review

Researchers are exploring ways to apply AI to improve education quality, enrich the learning process, enhance collaboration, tailoring, and motivation, and improve education’s collaboration, tailoring, and motivation. AI has also been applied to grading, learning analytics, and other aspects of education (Roll and Wylie, 2016). By utilizing AI, teachers’ evaluations may become more efficient, objective, and reliable, increasing the quality of the evaluation. An organization’s human resources (HR) plays an important role in mobilizing and coordinating additional resources to achieve its objectives. Effective management of HR within organizations and businesses is essential for developing reliable human resources. Performance evaluations provide a means of managing employee performance. Efficacy, quality, quantity, and effectiveness of an employee determine their performance and contribute to the organization’s success. However, it is important to keep in mind that the ability of teachers to achieve predetermined goals is a factor that can be considered part of the teachers’ performance (Budhwar et al., 2022; Putra et al., 2022).

The primary evaluation criteria shared among all educational systems worldwide include teacher competence, student interaction, relationships, personal characteristics, and content quality. There are, however, some differences in practical implementation and local application (Flores and Derrington, 2018). This is primarily because each country has different policies, procedures, and societal objectives.

CV, DL, ML, IoT, and other advanced tools may be applied to monitor, analyze, and enhance classroom dynamics using AI. Using AI technologies in classroom settings can significantly enhance student engagement and learning by allowing more profound insights into student interactions. However, it should be approached with caution, as there are privacy and ethical implications (Guo et al., 2021). In 2023, Li et al. developed an AI-based system to monitor, recognize, and analyze student behavior in real time by detecting interactions and capturing student participation, which enables educators to accommodate their teaching methods. The study included a multimodal dataset from real classrooms, such as video, audio, and sensor readings. The detected features were gestures, facial expressions, body poses, physiological metrics, participation, and speech patterns. A Convolutional Neural Network (CNN) was used to extract visual features, while recurrent neural networks (RNNs) or long short-term memory networks (LSTMs) were applied to capture temporal behavior patterns, and a Natural Language Processing (NLP) algorithm analyzed classroom conversations. For simple behavior detections, such as hand-raising and speaking, the accuracy was over 90%, while for more complex behaviors, the accuracy was between 75 and 85% (Li Y. et al., 2023).

Moreover, Li et al. examined the integration of AI and embedded devices in smart classrooms. Providing teachers with real-time feedback on student engagement and behavior helped enhance educational outcomes by enabling them to adjust their teaching strategies accordingly. Data collected by embedded devices includes information on visual, audio, and environmental characteristics. The studied extracted features include speech patterns, facial expressions, environmental factors, and body movements. The study employed several algorithms, such as CNNs, NLP, RNNs, and LSTMs. It illustrated the effectiveness of AI-based systems in accurately identifying student behaviors while providing teachers with immediate feedback. The results showed that the classroom teaching effect increased by 9.44% (Li L. et al., 2023).

A YOLOv8-based real-time monitoring system was developed by Chen et al. in 2023 to detect student behavior in the classroom. Students’ engagement, participation, and attentiveness are tracked and analyzed for educators to gain insights that will enable them to adapt their teaching methods as appropriate. The dataset employed includes video recordings from real classrooms. The extracted features included raising hands, standing, sitting, and interacting with learning materials. Results indicate that mean Average precision calculated at an intersection over union (IoU) threshold of 0.5 (mAP@0.5) has increased by 4.2 and 2.1%, respectively, indicating an improvement in object detection accuracy (Chen et al., 2023).

Moreover, using DL techniques, in 2023, Trabelsi et al. (2023) developed a system to monitor students’ attention levels in real-time. Students’ attention is intended to be maintained and improved by providing teachers with insights into their behavior, enabling them to adapt their teaching methods accordingly. The datasets include video recordings taken from real classrooms. Several features that indicate a student’s level of attention were extracted: facial expressions, head movements, eye gaze, posture, and eye movements. The study utilizes different models of the YOLOv5 algorithm, and the results demonstrate promising performance with 76% average accuracy (Trabelsi et al., 2023).

A recent study introduced by Ouyang et al. (2023) examined the use of AI-driven performance prediction combined with learning analytics in online engineering education to enhance student outcomes by enabling teachers to deliver more targeted assistance to students who are at risk. The dataset included personal information, logs of interactions, grades for assignments, quiz scores, and participation in discussion forums. The extracted features encompass student participation metrics, student performance indicators, and involvement in activities. This study combined an AI performance prediction model with learning analytics approaches to improve the learning effects in a collaborative learning context. The quasi-experiment demonstrated that the integrated approach increased student participation, boosted collaborative learning performance, and increased student satisfaction with learning (Ouyang et al., 2023).

In 2022, Bai et al. developed an intelligent system for evaluating the behavior of teacher-student interactions in the classroom based on the YOLO algorithm for recognizing and detecting objects. The dataset comprises video recordings from the smart classroom, with extracted features including student faces, the blackboard, head-up, and head-down positions. Teacher-student classroom behavior is recognized with an average accuracy of more than 90% for multiple classroom behaviors (Bai et al., 2022). The study by Khan et al. in 2021 investigated the development of an AI-driven system for monitoring student performance in real-time and devising preventive measures to improve academic outcomes. Students’ academic records for a course taught at Buruimi University College in the Sultanate of Oman were included in the dataset. They included ten features: gender, attendance, major, year, session, grades, CGPA, sponsorship, etc. The dataset was used to train several machine-learning algorithms: k-nearest Neighbors (k-NN), Decision tree, Multilayer Perceptron (MLP), Artificial Neural Networks (ANN), and Naïve Bayes. As a result, the decision tree came out on top with an accuracy rate of over 86% (Khan et al., 2021).

A sophisticated evaluation framework has been developed by Guo et al. (2021) to assess teaching effectiveness in the classroom using AI techniques that combine statistical modeling with ensemble learning to provide detailed insights into teaching quality and student engagement. The dataset includes the recording of the classroom, teaching observations, interaction records, student feedback surveys, and academic performance metrics. An analysis of the data includes learning practices, levels of student engagement, performance data, and feedback from surveys and observations. Statistical techniques were utilized, including multiple linear regression and factor analysis, and ensemble learning techniques such as Random Forests (RF) and Gradient Boosting Machines (GBMs) to enhance the evaluation’s quality. The results demonstrate that the student’s concentration and participation achieved an accuracy of 8.318 and 9.375, while teachers’ media usage and teachers’ type had an accuracy of 0.905 and 0.815. It is estimated that ensemble learning can evaluate teachers’ style with an accuracy of 0.73, higher than the statistical modeling module with an accuracy of 0.69 (Guo et al., 2021).

The literature review confirms that AI-based systems can accurately detect classroom behaviors and interactions. This is accomplished by exploiting several AI techniques, such as ML algorithms, CV, and NLP. This work aims to introduce a model for detecting student-teacher interactions, interpret it for easy understanding, and provide comprehensive recommendations to ensure the effectiveness of the system’s implementation.

Regarding CV, there are numerous classic methods for object detection. As a milestone in developing DL-based object detection, Girshick et al. (2014). proposed the R-CNN. A region candidate box was selected instead of a sliding window to traverse over a picture, which might contain the objects to be detected. Five convolutional layers and two fully connected layers were included in the R-CNN architecture. Further improvements to the R-CNN were achieved by SPP-Net (He et al., 2015), Fast R-CNN (Girshick, 2015), Faster R-CNN (Ren et al., 2016), R-FCN (Dai et al., 2016), and Mask R-CNN (He et al., 2017). According to Redmon (2016) the YOLO algorithm differentiates between object detection algorithms: one-stage such as YOLOv2 (Redmon and Farhadi, 2017), YOLOv3 (Redmon and Farhadi, 2018), SSD (Liu et al., 2016), and RetinaNet and two-stage algorithms such as Faster R-CNN (Kaur and Singh, 2023). The primary contrast is that YOLO discarded the candidate box extraction branch. A branchless convolutional network was used in the YOLO algorithm to extract features, simulate candidate frames, and classify them, thereby simplifying the network structure, resulting in a detection speed almost ten times faster than Faster R-CNN (Yang et al., 2020).

Table 1 summarizes the related works regarding objectives, studied features, and algorithms used, allowing us to compare them with the proposed work. As shown in the following table, all works focus on monitoring, tracking, recognizing, and analyzing the behaviors and activities of students in the classroom. It should be noted that similar to ours, some studies focus on the interaction between students and teachers, such as (Bai et al., 2022; Guo et al., 2021). Additionally, our study’s purpose is to detect interactions in the classroom and thus reflect the result on one aspect of evaluating teacher performance. Most other studies focused on assessing teaching effectiveness through metrics such as student behavior, participation, attention, and engagement to enhance students’ outcomes and the quality of the teaching process. Furthermore, unlike the rest of the studies, the proposed study and (Ouyang et al., 2023; Bai et al., 2022; Guo et al., 2021) did not focus on real-time interactions, as their systems rely on real-time feedback to adjust teaching methods.

**Table 1:** Related works summary.

N	Authors	Objective	Targeted object/s	Real-time	Studied features	Dataset	Utilized algorithms	Results (Accuracy)
1	Li Y. et al. (2023)	Adjusting teaching methods	Students	Yes	Gestures, facial expressions, body pose, physiological metrics, participation, and speech patterns	Video, audio, and sensor readings	CNN, RNNs, LSTMs, and NLP	For Simple Behaviors: >90% for complex Behaviors: 75–85%
2	Li L. et al. (2023)	Adjusting teaching strategies	Student	Yes	Speech patterns, facial expressions, environmental factors, and body movements.	Visual, audio, and environmental characteristics	CNNs, NLP, RNNs, and LSTMs	Teaching effect increased by 9.44%
3	Chen et al. (2023)	Adapting teaching methods	Student	Yes	Raising hands, standing, sitting, and interacting with learning materials	Video recordings from real classrooms	YOLOv8	76.3%
4	Trabelsi et al. (2023)	Adapting teaching methods	Student	Yes	Facial expressions, head movements, eye gaze, posture, and eye movements	Video recordings from real classrooms	YOLOv5	76%
5	Ouyang et al. (2023)	Enhancing student outcomes	Student	No	Personal information, interaction logs, scores, and participation in discussion forums.	Students’ performance metrics (Text Records)	AI performance prediction model	No detection results
6	Bai et al. (2022)	Improving teaching quality	Student and Teacher	No	Face students, face blackboard, head up, and head down	Video recordings from real classrooms	YOLO	90%
7	Khan et al. (2021)	Improving academic outcomes	Student	Yes	Gen, attendance, major, year, session, grades, CGPA, sponsorship	Student’s academic records (Text Records)	k-NN, Decision tree, MLP, ANN and Naïve Bayes	86%
8	Guo et al. (2021)	Evaluating teaching effectiveness	Student and Teacher	No	Students’ concentration, students’ participation, teachers’ type, teachers’ style, and teachers’ media usage.	Classroom recording, interaction records, feedback surveys, and academic metrics	Statistical techniques and ensemble learning techniques	81%
9	Proposed work	Evaluate teachers’ performance	Student and Teacher	No	Using the textbook, students’ participation, teacher’s activities, and students’ activities	Video recordings from real classrooms	Yolov8, Detectron2: Faster R-CNN, and RetinaNet	YOLOv8x: 85.8% Faster R-CNN: 72.7% RetinaNet: 70.6%

Even though most studies have used video recordings of classrooms to extract the features, some have also included audio recordings, sensor readings, and academic data (mainly textual). Approximately thirty features were studied; however, in this work, we only highlight four main elements and eleven sub-elements that can be used to evaluate teacher performance related to classroom interactions. Regarding AI-based techniques utilized in the previous works, they varied between NLP (Li Y. et al., 2023; Li L. et al., 2023; Guo et al., 2021), CV (Li Y. et al., 2023; Li L. et al., 2023; Chen et al., 2023; Trabelsi et al., 2023; Ouyang et al., 2023), statistical analysis (Ouyang et al., 2023; Guo et al., 2021), and ensemble DL (Guo et al., 2021). Our proposed research focuses on CV, particularly object detection. We utilize three leading object detection algorithms known for their accuracy and speed.

Compared to prior research, which often concentrated on student-focused metrics such as participation, attention, or academic outcomes, our study uniquely emphasizes teacher evaluation through observed student-teacher interactions. Unlike studies that broadly monitor behavior or rely on multimodal data, our work offers a focused application of object detection techniques using a custom-labeled dataset representing eleven specific interaction categories. While several existing systems have employed CNNs or YOLO-based approaches for behavior tracking, our contribution lies in the integration of multiple state-of-the-art object detection models—YOLOv8, Faster R-CNN, and RetinaNet—within a unified framework. This comparative setup not only benchmarks model performance in detecting educational interactions but also advances the field by aligning detection output with teacher performance evaluation metrics. This focused framework addresses the gap in research that connects object detection outputs to real pedagogical evaluation criteria.

## 3 Materials and methods

This study aims to address the challenges associated with traditional teacher performance evaluation by focusing on one of the Teacher Key Performance Indicator (T-KPI) elements identified in (Almubarak et al., 2024) namely, the teacher’s proficiency in presenting lessons and managing the classroom. To that end, we propose a Computer Vision (CV) and Deep Learning (DL)-based system that detects teacher–student interactions in the classroom. The system leverages object detection algorithms, including YOLO (You Only Look Once), Faster R-CNN (Faster Region-based Convolutional Neural Network), and RetinaNet. The methodology section outlines the classification levels for four key types of interactions: textbook usage, student participation, student activities, and teacher activities.

**Figure 1:**
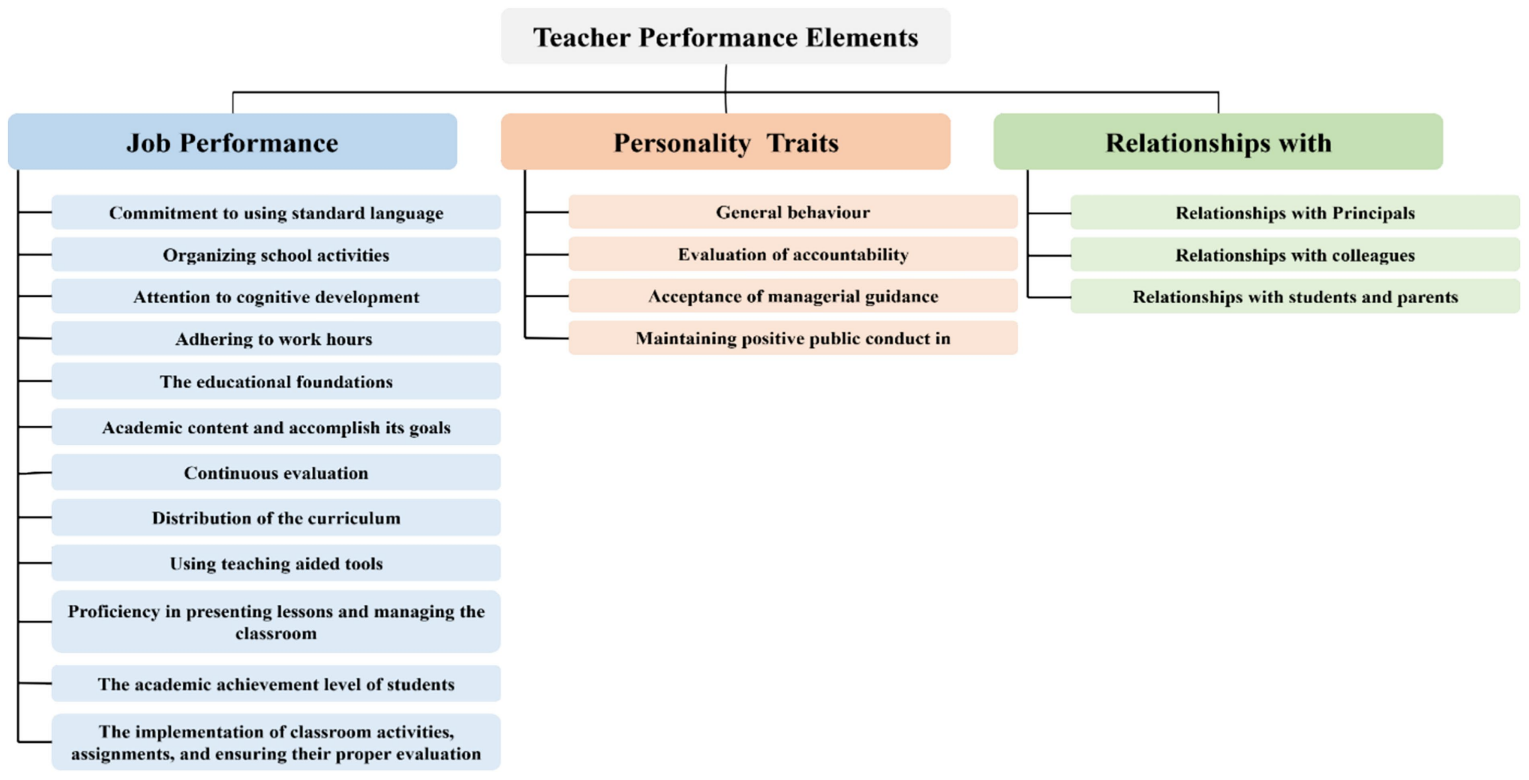
Analyzing the KPIs of teacher performance evaluation in Saudi Arabia.

The analytic evaluation index introduced by (Almubarak et al., 2024) and adopted here contains three main evaluation elements: Job Performance, Personal Traits, and Relationships. A total of nineteen sub-evaluation elements is under the main elements: Job Performance includes 12, Personality Traits contains 4, and Relationships addresses three sub-elements, respectively as shown in Figure 1. Further, the main and sub-element analysis resulted in ninety-nine detailed elements https://tinyurl.com/236h7xve. Each detailed element represents an indicator that can be utilized to evaluate each sub-element. However, we are specifically addressing the tenth sub-element, which relates to the teacher’s proficiency in presenting lessons and managing the classroom, as the element under study. The evaluation involves three elements of the educational process: the student, the teacher, and the educational environment, making it highly complex. In addition, three AI techniques may be utilized: IoT, CV, and speech processing. This evaluation focuses on three detailed elements: (a) teaching methods, (b) classroom interactions, and (c) seating arrangement based on educational principles. Among these, classroom interactions were identified as the most crucial, as the other elements rely on their effectiveness. Furthermore, it provides a valuable indication of the quality of the introduced content and the level of student engagement.

We have employed three widely used object detection algorithms to develop DL models, YOLOv8, Faster R-CNN, and RetinaNet, to enhance accuracy, robustness, and generalization and reduce bias and variance. A real-time object detection model designed for real-time performance, YOLOv8 is the latest in the YOLO series (Terven et al., 2023). In this approach, the image is processed in only one forward pass, making it suitable for applications requiring speed. In addition, YOLOv8 introduces architectural improvements such as an optimized backbone for better feature extraction and a neck (using Feature Pyramid Networks or Path Aggregation Networks) to enhance object detection at various scales. An anchor-free detection head simplifies the detection process by directly predicting bounding box coordinates, object classes, and confidence scores from the model. A non-maximum suppression (NMS) technique is used to refine the final predictions by eliminate redundant findings.

Faster R-CNN is built on the R-CNN family and is a two-stage model for object detection (Ren et al., 2016). Due to its high level of accuracy, it is perfect for scenarios in which precision is of high importance. Faster R-CNN uses a backbone like ResNet or VGG to extract features. Region Proposal Networks (RPN) are the first stage of the process. RPNs are crucial innovations used to predict regions likely to contain objects, thereby improving the efficiency and speed with which the regions are proposed. The RPN outputs object scores and bounding boxes using a sliding window approach on feature maps. The second stage consists of applying ROI Pooling to pool regions into a fixed size, followed by applying fully connected layers to classify objects and carry out bounding box regression. Even though it is slower than single-stage detectors like YOLO, this multi-step approach ensures accurate object detection.

RetinaNet addresses class imbalances between small objects and the background during object detection (Tan et al., 2021). Feature Pyramid Networks (FPNs) are integrated with ResNet backbones for feature extraction to improve the capability of the system to detect objects at various scales. Through FPN, RetinaNet can detect objects of varying sizes because feature maps are merged from different layers within the network. Among RetinaNet’s noteworthy innovations is its focal loss function, which reduces the impact of easy-to-detect background objects and increases the concentration of difficult-to-detect targets. The implementation of this algorithm dramatically improves the performance on datasets with class imbalances, where certain classes (especially background classes) dominate the rest of the dataset. RetinaNet uses anchor boxes like other object detectors but incorporates focal loss to better handle imbalances between foreground and background objects. Object classes are predicted, and bounding boxes are refined using the classification and regression heads applied to the FPN.

RetinaNet was designed to balance the high speed of one-stage detectors such as YOLO and the accuracy of two-stage detectors such as Faster R-CNNs, making it suitable for tasks that require both efficiency and quality detection. YOLOv8 excels in tasks requiring fast reaction times, Faster R-CNN in tasks demanding high precision, and RetinaNet balances speed and accuracy while addressing class imbalance (Tan et al., 2021).

Beyond object detection, the models in this study also perform classification across eleven distinct behavioral categories that capture key classroom interactions linked to teacher performance. These classification outcomes are not viewed as the final product, but rather as foundational inputs for a follow-up study currently in progress. In the next phase, a detailed analysis of category-wise performance will be conducted to identify behaviors with low representation or those that are semantically overlapping. Insights from this analysis will guide the refinement of class definitions—through either the removal of low-impact categories or the merging of similar ones—to develop a more streamlined, high-accuracy scoring framework for teacher performance evaluation.

### 3.1 Proposed teacher performance evaluation framework

**Figure 2:**
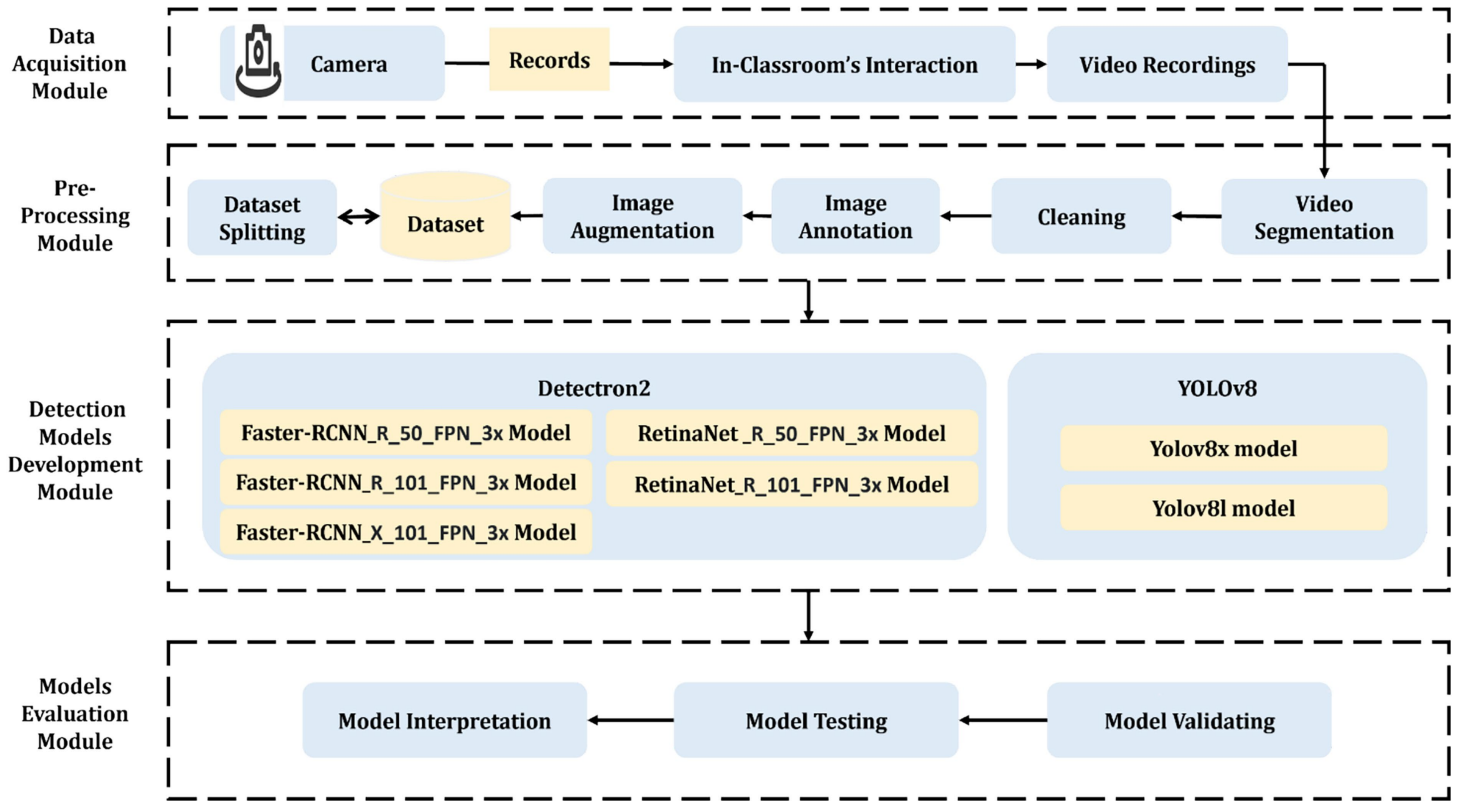
The general framework of the proposed system.

The proposed system’s methodology, depicted in Figure 2, outlines a comprehensive teacher performance evaluation framework composed of four interconnected modules. The Data Acquisition Module initiates the pipeline by capturing in-classroom video recordings using fixed-position high-resolution cameras, forming the raw input for the system. These videos are then processed by the Pre-Processing Module, which includes sequential stages: video segmentation (converting videos into individual frames), cleaning (removing noisy or irrelevant frames), annotation (labeling interactions using Roboflow), image augmentation (enhancing diversity through rotation, saturation, and noise), and finally, dataset splitting into training, validation, and test sets.

This processed dataset is passed to the Detection Models Development Module, where two state-of-the-art frameworks—YOLOv8 and Detectron2—are applied using multiple variants (e.g., YOLOv8x, RetinaNet, Faster R-CNN). These models are trained to detect and classify eleven fine-grained classroom interaction behaviors across four main interaction categories. Finally, the Models Evaluation Module interprets the trained models’ performance using several evaluation metrics (e.g., mAP, Precision, Recall, IoU), highlighting their accuracy, robustness, and readiness for deployment. Each module produces intermediate results—such as the number of segmented frames, the reduced dataset size after cleaning, and per-class detection accuracy—which are used to assess and refine the system’s overall effectiveness.

#### 3.1.1 Data acquisition module

The study’s data collection environment included the primary and intermediate levels of QURTUBAH Private Schools affiliated with the Jeddah Education Department. A high-resolution camera was mounted to capture classroom interactions, but multiple cameras may be required to cover a larger classroom. The videos captured for classes of different subjects introduced by different teachers amounted to about eighteen videos. The subjects included science, mathematics, English, Arabic, and social studies.

Measures have been taken to protect the privacy rights of students and teachers. Initially, to ensure the legality of the data collection process, we obtained official approval from the Ministry of Education in the Kingdom of Saudi Arabia, the Education Department in Jeddah, and the owner and principal of QURTUBAH Private Schools. Secondly, informed consent was obtained from participants by the school administration, which was accomplished by explaining clearly the purpose, the methodology, and data access permission. Moreover, our research team strictly adheres to all relevant privacy and data protection laws, including Saudi Arabia’s regulations. By implementing these measures, we sought to safeguard the privacy rights of our participants and ensure that our research was ethical and responsible. Responsible.

#### 3.1.2 Pre-processing module

This module has five phases: video segmentation, image normalization, annotation, augmentation, and dataset splitting. The collected videos were processed using Roboflow (Roboflow, 2024), a powerful CV tool for better data collection, preprocessing, and model training techniques. The videos were segmented into frames at a rate of one frame every 3 s. This rate was chosen after testing multiple intervals (five, six, eight, and 10 s), as higher intervals risked missing brief but critical classroom behaviors such as raising hands (participation). The segmentation process produced 7,008 frames, which were cleaned by excluding those with high noise that obscured the studied features. This cleaning step ensured that only high-quality frames were passed to the annotation and training pipeline, strengthening the reliability of model learning. Roboflow also assigned multiple labels for each image during the image annotation process to support effective object detection model training.

Following annotation, the dataset was automatically oriented and resized to 224 × 224 pixels. An image augmentation process was then applied to improve model performance and generalization. Specifically, the images underwent 15-degree rotation, a 25% increase in saturation, and 1.02% added noise, expanding the dataset to 7,259 images. Finally, the dataset was split into 70% for training, 15% for validation, and 15% for testing, resulting in 6,369 training images, 452 validation images, and 450 test images. Moreover, the final dataset includes a total of 31,265 labels.

#### 3.1.3 Classification of the studied features

**Figure 3:**
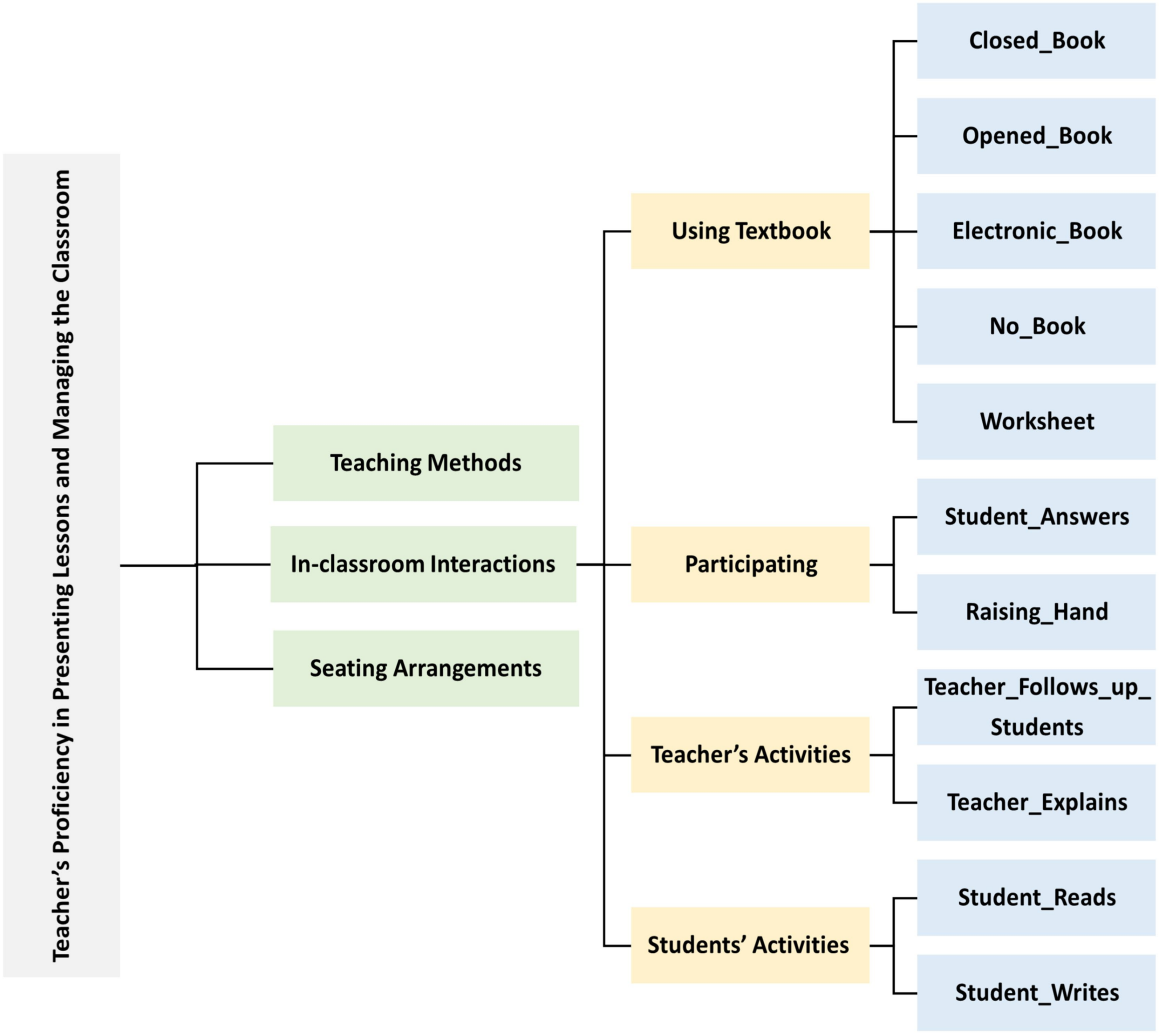
Classification of in-classroom interactions.

Automatic detection of classroom interactions allows school principals to assess one aspect of a teacher’s proficiency in presenting lessons and managing the classroom from the 19 elements of the teacher performance assessment. This enables the identification of the strengths and weaknesses of a teacher’s competence. This study addresses four main, and eleven sub-features of activities associated with in-classroom interactions to train our models, as illustrated in Figure 3. These features include using a Textbook, Participating, Teacher’s Activities, and Students’ Activities. These eleven subcategories were used to label and classify classroom interactions in the dataset, forming the basis for model training and later evaluation. Five textbook usage levels were considered: Open Book, Closed Book, Electronic Book, No Book, and Worksheet. Regarding students’ participation, we considered two levels: Raising Hands and Answering. As for activities, we considered four levels two for both teachers and students: Teacher Follows up Students, Teacher Explains, Student Reads, and Student Writes.

**Figure 4:**
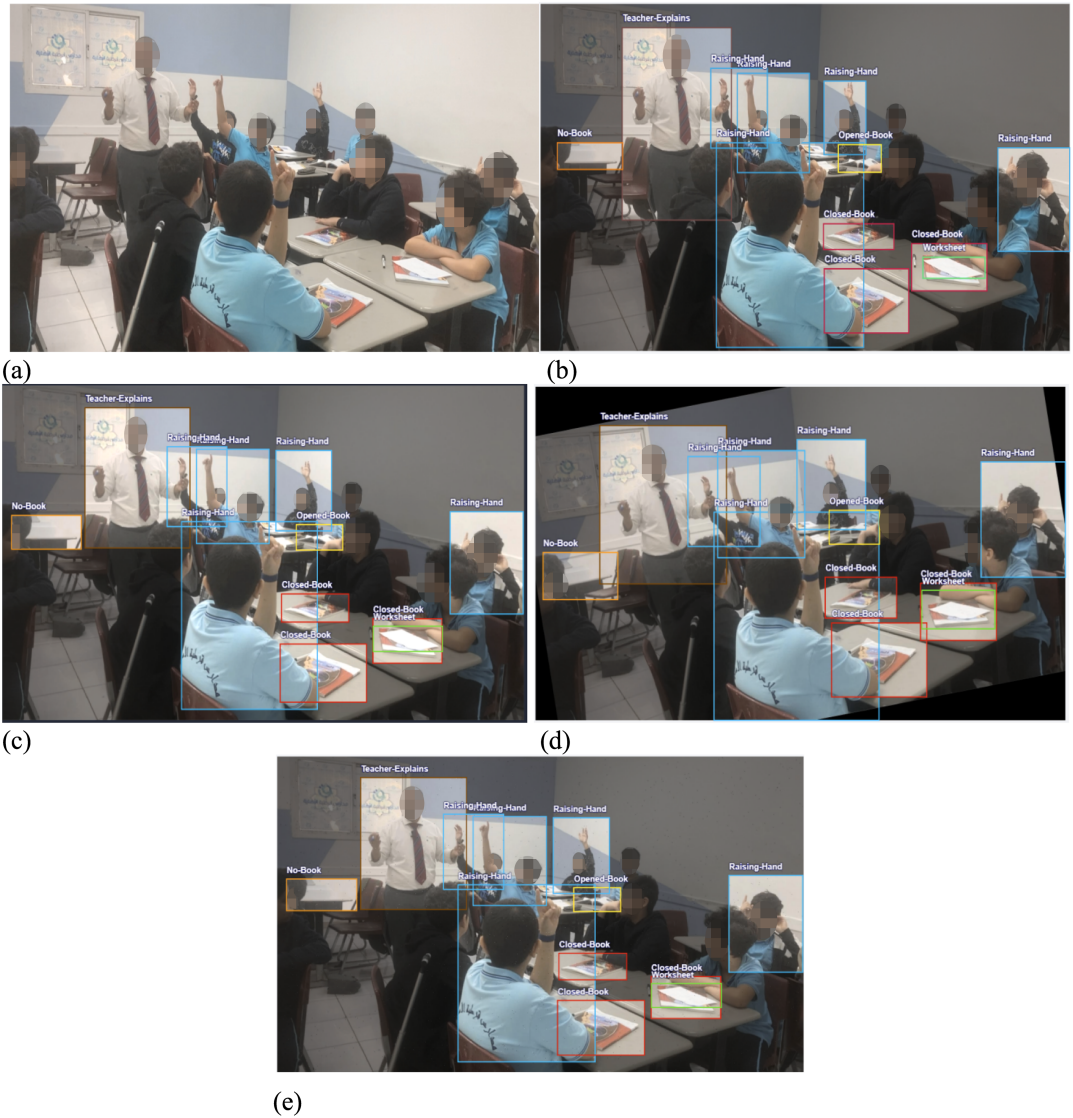
Example of data transformation from raw to augmented images **(a)** Raw Image, **(b)** Image with Annotation, **(c)** Augmented Image_Saturation, **(d)** Augmented Image_Rotation, **(e)** Augmented Image_Noise.

**Figure 5:**
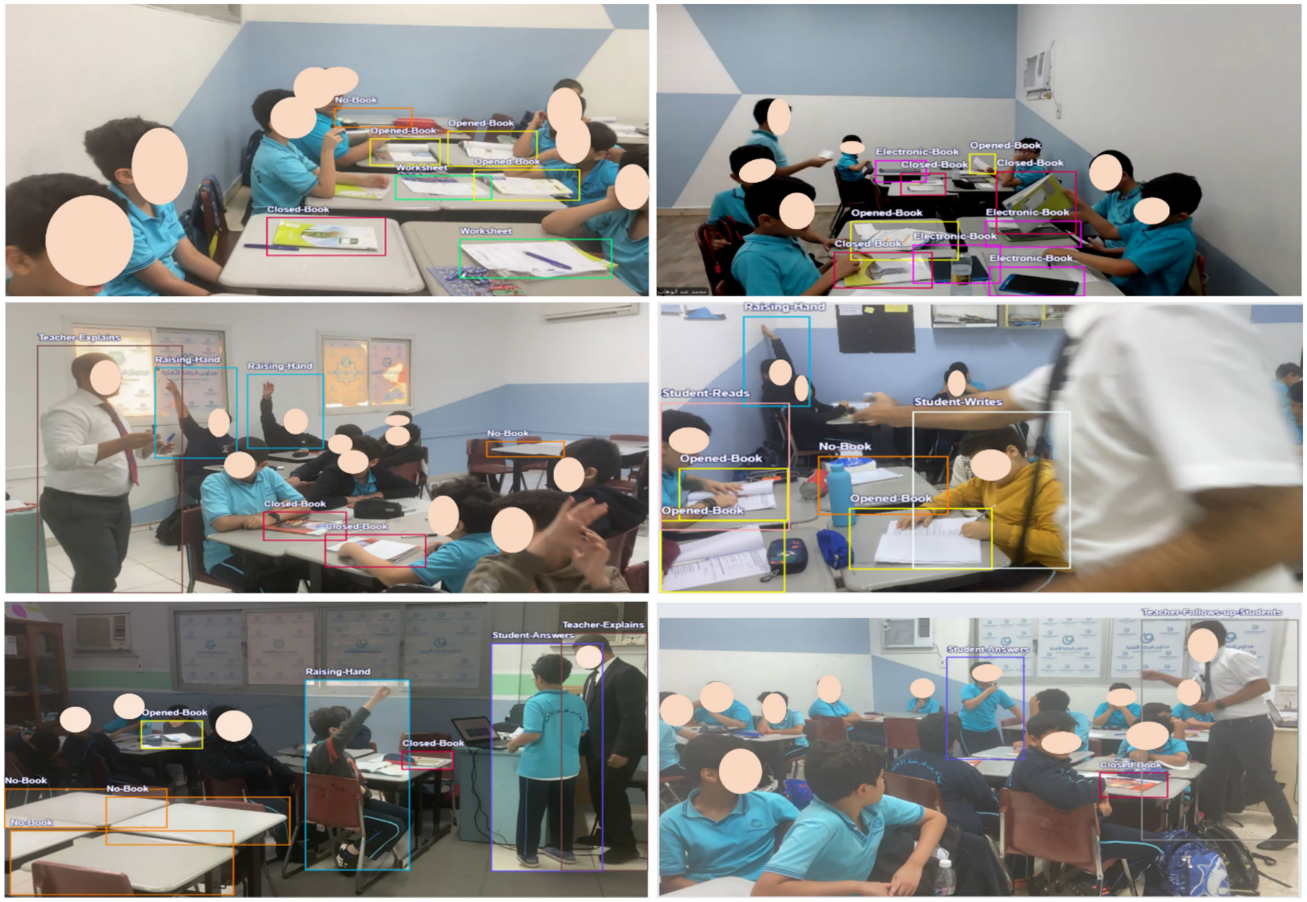
Sample images for in-classroom interactions dataset.

To determine the indicators of the states (levels) of interaction within the classroom, we studied each category separately based on our previous in-depth analysis of the T-KPIs (Almubarak et al., 2024), as illustrated in Table 2. It also shows the count of each label in the dataset. For instance, the label ‘Closed Book’ appeared in 1210 images and 2,147 times. Moreover, some of the image samples are shown in Figure 4. Moreover, Figure 5 illustrates the data augmentation flow, demonstrating the transformation of raw images into augmented versions. This visual representation highlights the various applied augmentation techniques, to enhance the diversity and robustness of the training dataset.

**Table 2:** Dataset description.

Classes (Labels)		Indicators	Number of instances
Using_Textbooks	Closed book	Closed book in the student’s hand Closed book on the table	2,147
	Opened book	Opened book in the student’s hand Opened book on the table	4,547
	Electronic book	Tablets on the table Tablets in the student’s hand	124
	No book	A table without a textbook or e-book	2,206
	Worksheet	Worksheet in the student’s hand Worksheet on the table	781
Participation	Raising hand	Student raising hand standing or sitting	1,450
	Student answers	Student standing in the classroom Student writing on the board	417
Teacher’s activities	Teacher explains	Teacher standing in front of the blackboard	455
	Teacher follows up students	A teacher walks among the students A teacher leans forward to read and follow what the students are writing	73
Student’s activities	Student reads	Student’s eye direction to the book Student’s holding of the book	435
	Student writes	Student holding pen Student writing on book or worksheet	323

#### 3.1.4 Models development module

Our proposed models are trained, validated, and tested by using Google Colab (Bisong, 2019), a hosted Jupyter Notebook service (Kluyver et al., 2016) that provides access to cloud-based computing resources, including GPUs and TPUs. We have employed three widely used object detection algorithms to apply the DL models: YOLOv8, Fast-er-RCNN, and RetinaNet. The source code of our proporsed models is available on Gitbub https://github.com/ArwaASM/In-classroom-Interaction-Detection

Regarding YOLOv8, five further versions are available, ranging from nanoscale to extra-large models. When selecting these models, evaluating the tradeoff between accuracy required and inference time is necessary. For in-classroom interaction behavior detection, we do not target real-time applications; thus, the accuracy is more significant than speed in this case. We utilized the large and extra-large versions of YOLOv8. The models were trained for 120 epochs with a batch size of 16 and an image size of 800, subject to GPU memory constraints. The learning rate used during model training was 0.01, with an SGD momentum of 0.937 and an optimizer weight decay of 0.0005. All other training parameters used the YOLOv8 network’s default values.

**Figure 6:**
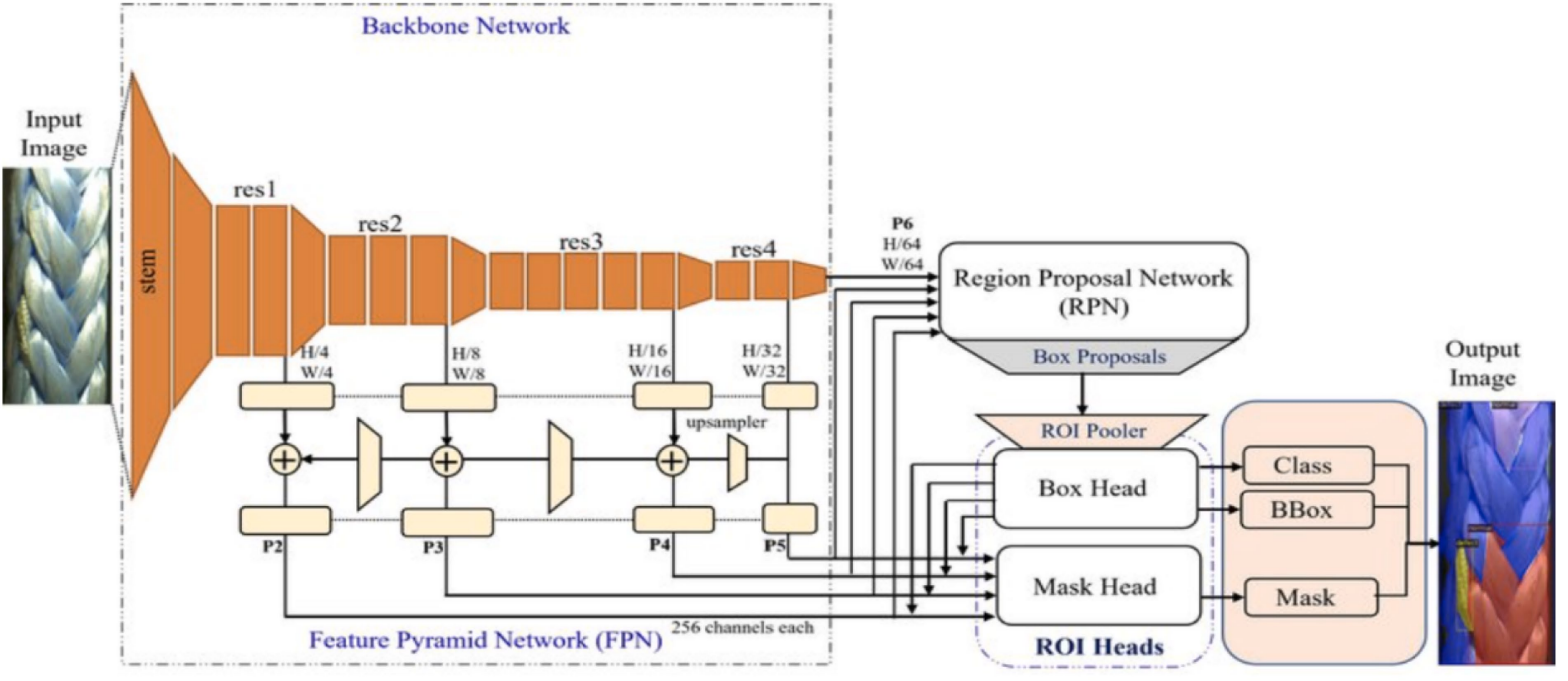
Detectron2 architecture. *Input Image*: The original raw image provided to the model. *Stem*: The initial convolutional layers that prepare the image features. *Backbone Network (res1–res4)*: A sequence of layers that extract hierarchical feature maps from the input image. H/W (Height/Width) annotations: Indicate the spatial resolution reduction at each stage. *Feature Pyramid Network (FPN)*: Combines multi-scale feature maps (P2–P6) for detecting objects of different sizes. *Plus signs (+)*: Represent the merging of feature maps from different layers. *Upsampler*: Increases the resolution of feature maps before merging. *Region Proposal Network (RPN)*: Generates candidate bounding boxes (box proposals) from feature maps. *Solid arrows*: Show the flow of data through the network components. *ROI Pooler*: Extracts fixed-size feature representations from box proposals. *Box Head*: Predicts object class labels and refines bounding boxes. Mask Head: Predicts segmentation masks for each detected object. *Class, BBox, Mask blocks*: Represent the output layers for class prediction, bounding box regression, and mask generation, respectively. *Output Image*: The final image displaying detected objects with bounding boxes, class labels, and instance masks.

To implement Faster R-CNN and RetinaNet, the Detectron2 framework (Wu et al., 2019), a CV model zoo written by the FAIR Facebook AI Research group, was used. It includes all the models that were available in the original Detectron, such as Faster R-CNN, Mask R-CNN, RetinaNet, and DensePose (Güler et al., 2018), as well as some newer models, including Cascade R-CNN, Panoptic FPN (Kirillov et al., 2019), and TensorMask (Chen et al., 2019). Moreover, as shown in Figure 6 the backbone, the neck, the region proposal network (RPN), and the head represent the primary components of Detectron2. The backbone uses various architectures, such as ResNet (He et al., 2016), ResNeXt, and MobileNet, to extract features from the input image. A large-scale image dataset, such as ImageNet, is often used to train these architectures. Several convolutional layers are organized hierarchically as the backbone of the network. By increasing the number of channels, these layers gradually reduce the spatial dimension of the feature maps. FPN is implemented as the neck component to refine the feature maps acquired from the backbone. FPN can detect objects of various sizes and scales by combining features from different scales into a multiscale feature pyramid (Ju and Cai, 2023).

Nevertheless, some DL-based object detectors, such as YOLO, do not contain this part and are called single-shot detectors. RPN generates approximately 1,000 box proposals with confidence scores after analyzing multi-scale features. Objects of interest within this image are represented by these potential bounding boxes. Additionally, Detectron2 employs a box head for cropping and wrapping feature maps into multiple fixed-size elements. Then, NMS filters out around 100 boxes.

From Detectron2, we have selected two main models: Faster R-CNN, which belongs to two-shot detectors, and RetinaNet, which belongs to single-shot detectors (Lin et al., 2017). To develop Faster R-CNN and RetinaNet algorithms, we adopted five models which are: Faster_R-CNN_X_101_32x8d_FPN_3x, Faster_R-CNN_R_101_FPN_3x, Faster_R-CNN_R_50_FPN_3x, RetinaNet_R_50_FPN_3x, and Reti-naNet_R_101_FPN_3x. The backbones of the chosen models were ResNet and Res-NeXt, with 50 and 101 layers. To refine the feature maps generated from the backbones, we used FPN neck. All these models have been trained for 3x the standard iterations, respectively. The training was set up to be trained for 100 epochs (39,850 iterations), with a batch size of 16, an image size of 800, and a 0.01 learning rate. All other training parameters were set to the default values of the Detectron2 framework.

#### 3.1.5 Model evaluation module

**Figure 7:**
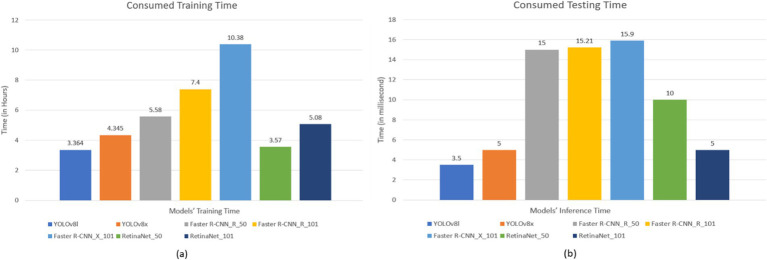
Models’ consumed time **(a)** Models’ training time (Hours) and **(b)** Models’ testing time (millisecond).

**Figure 8:**
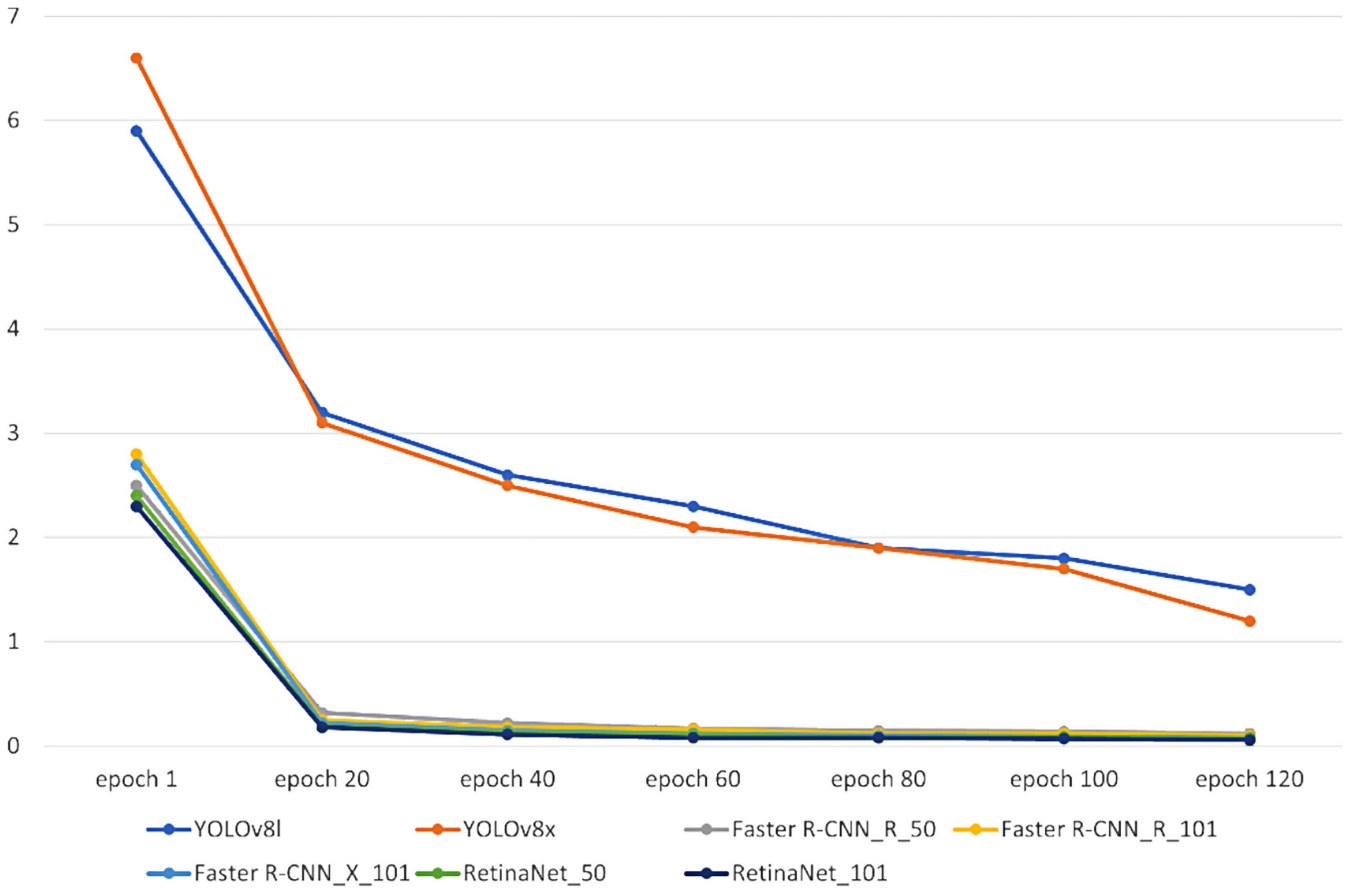
Models’ total losses.

**Figure 9:**
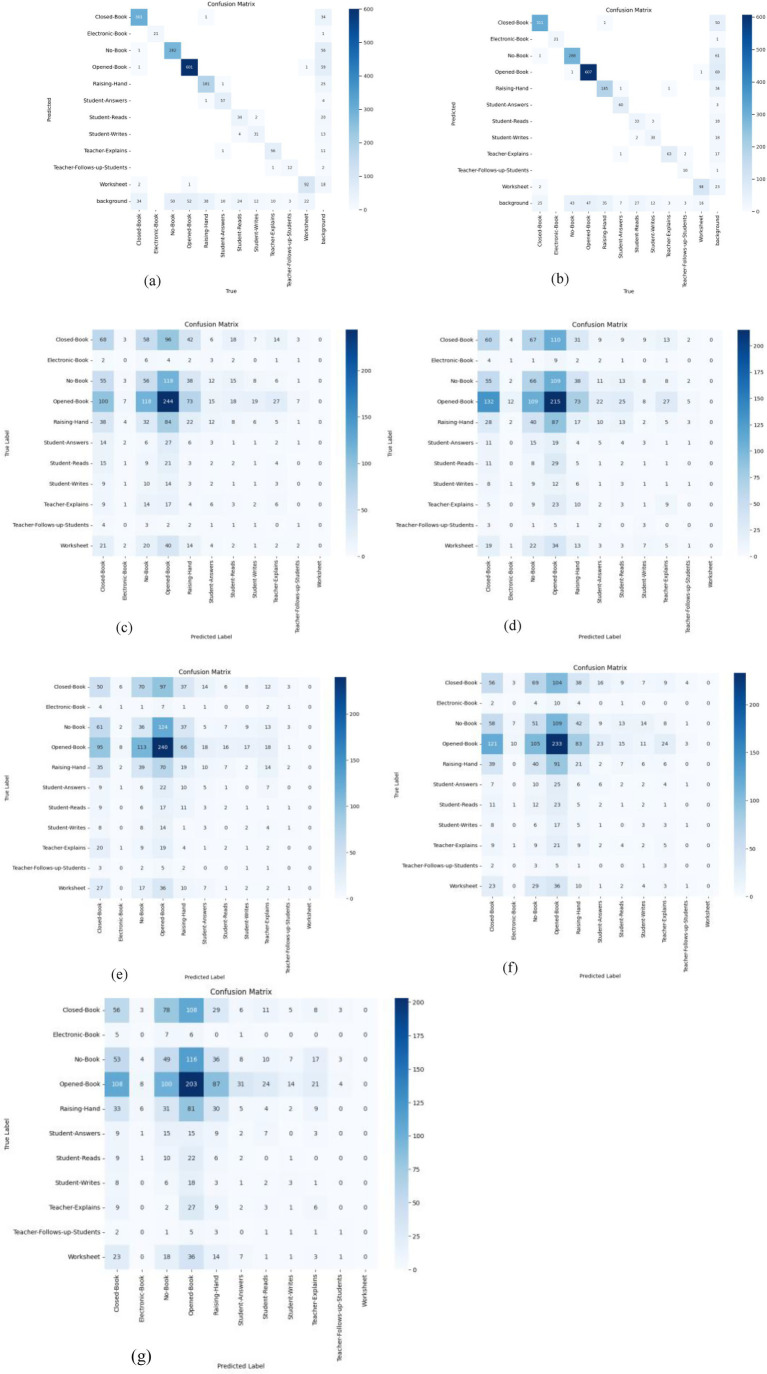
Models’ confusion matrices **(a)** YOLOv8x, **(b)** YOLOv8l, **(c)** Faster R-CNN_R_50, **(d)** Faster R-CNN_R_101, **(e)** Faster R-CNN_X_101, **(f)** RetinaNet_50, and **(g)** RetinaNet_101.

**Figure 10:**
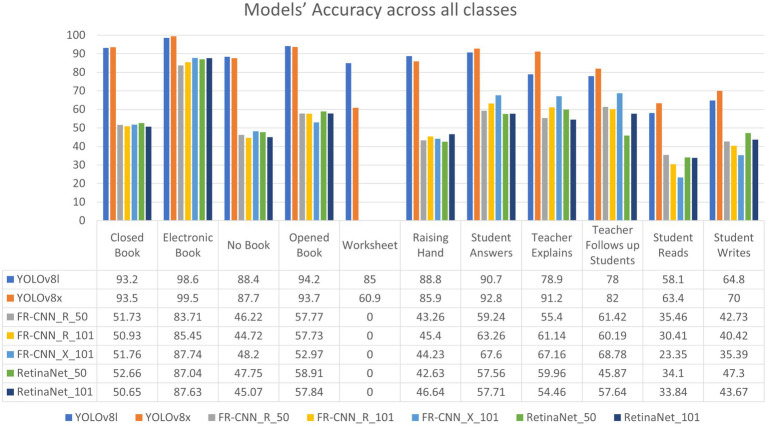
Models’ AP scores across the eleven classes.

**Figure 11:**
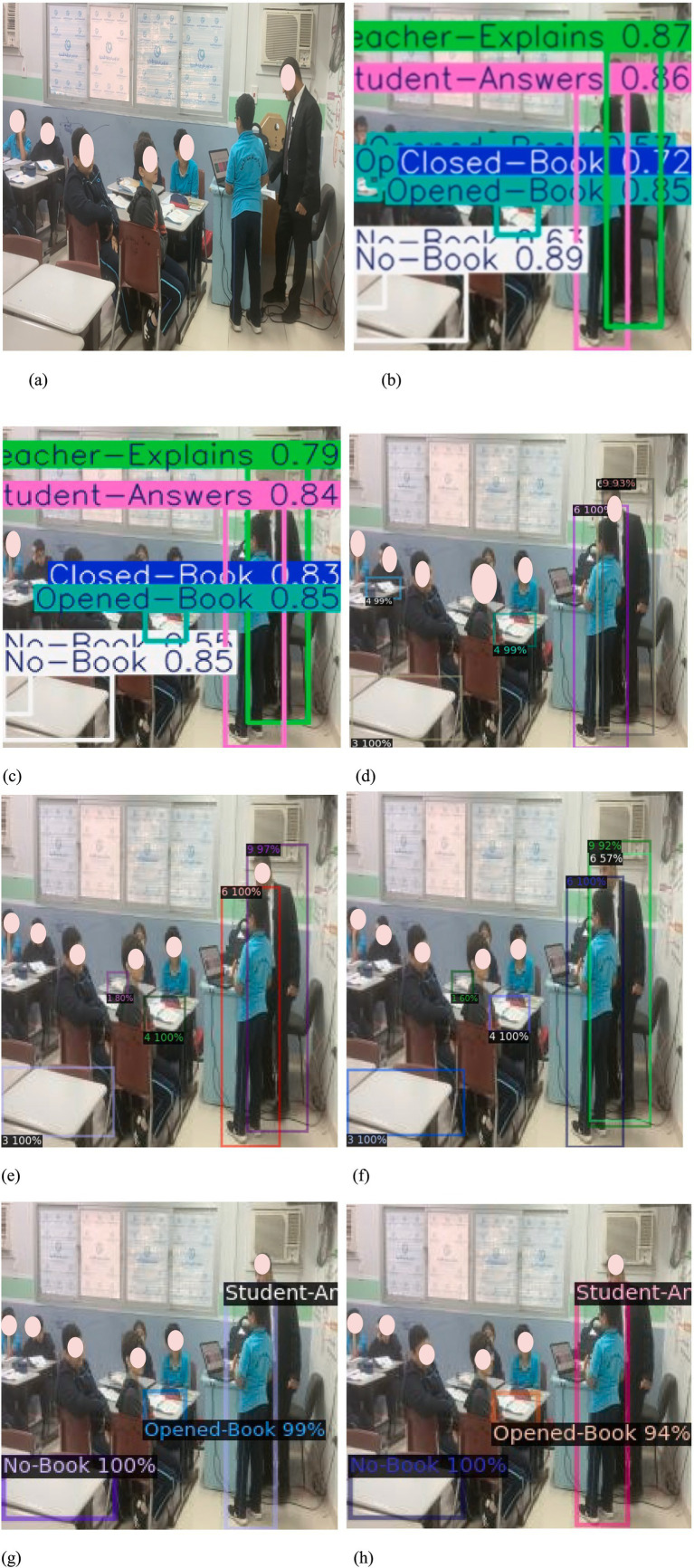
**(a)** Random sample from the testing dataset **(b)** YOLOv8l **(c)** YOLOv8sx **(d)** Faster R-CNN_50 **(e)** Faster R-CNN_101 **(f)** Faster R-CNN_x_101 **(g)** RetinaNet_50 **(h)** RetinaNet_101.

In this study, we utilized our in-classroom interaction dataset to train and evaluate the efficiency of interaction detection models. Object detection and localization accuracy must be measured systematically and objectively. The outputs of the object detection and classification stages are reported in Table 4 and Figures 7–11, where each model’s per-class accuracy, precision, and robustness are analyzed to assess their suitability for automated evaluation Thus, to assess the performance of the proposed model, we highlight several evaluation metrics that Yolo and Detectron2 generated. These metrics include mean average precision (mAP), Precision, Recall, Average Precision (AP), F1 Score, Box Loss, Distance-Focal Loss 1 (DF1 Loss), Intersection over Union (IoU), and Classification Loss (Cls Loss).

**Table 4:** Models’ accuracy.

Models	Training accuracy	Testing accuracy	
	Accuracy	mAP50	mAP50-95
YOLOv8l	85.3%	84.5%	58.9%
YOLOv8x	84.5%	85.8%	62.8%
Faster R-CNN_R_50	98.69%	72.7%	48.8%
Faster R-CNN_R_101	98.82%	71.4%	49.1%
Faster R-CNN_X_101	99.1%	72.4%	49.7%
RetinaNet_R_50	90.3%	70.5%	48.5%
RetinaNet_R_101	90.00%	70.6%	48.7%

Precision (P) indicates the percentage of correctly predicted objects (true positives) out of all predicted objects (accuracy), as shown in Equation (1).


(1) $$P=\frac{TP}{TP+FP}$$

Where TP represents the True Positive (the number of target frames correctly predicted to be in the positive category) and FP is the False Positive (the number of target frames incorrectly predicted to be in the positive category).

Recall (R) measures the percentage of actual objects the model successfully detects, showing its ability to find all relevant objects, as shown in Equation (2).


(2) $$R=\frac{TP}{TP+FN}$$

Where FN represents the false negatives (the number of target frames in the positive category but incorrectly predicted to be in the negative category).

Averaging precision (AP) across recall levels summarizes the precision-recall curve, as shown in Equation 3. It provides a per-class measure of detection performance. Additionally, detectron2 presents other AP metrics, such as AP75, APl, APm, and APs. In AP75, precision is measured at different recall values, and TP is determined when the IoU overlap is greater than 75%. The accuracy of APl is calculated at different recall values for objects with a large area (area > 952). In contrast, APm computes the precision at various recall scores for medium-sized objects (322 > area > 962). For small size objects, APs calculate precision based on a different recall value (area x 322).


(3) $$AP=\int_{0}^{1}PRdr$$

mAP measures the average AP scores across all object classes shown in Equation 4, mAP gives an overall evaluation of the model. For object detection models, two threshold values are commonly used: the mAP50 (the mean of AP based on confidence scores between 0 and 0.50) and the mAP50-90 (the mean of AP based on confidence scores between 0.50 and 0.95) (Dang et al., 2023).


(4) $$\;mAP=\frac{1}{n}\sum_{i=1}^{n}AP_{i}$$

IoU indicates the overlap between the predicted and ground truth bounding boxes, divided by the sum of the unions as shown in Equation 5; a higher IoU means better bounding box accuracy.


(5) $$IoU=\frac{AreaofOverlap\;}{AreaofUnion}\;$$

Box Loss (Localization Loss) measures the difference between the predicted bounding box coordinates and the ground truth box. Loss functions such as Smooth L1 or IoU-based losses are used to optimize localization accuracy, as shown in Equation 6.


(6) $$Box\;Loss=Smooth\;L1\;\left(pred_{box},\;\;gt_{box}\right)$$

DF1 Loss is a distance-based loss that emphasizes harder-to-detect objects by applying higher penalties to samples challenging to classify or localize. This loss is beneficial for boundary-sensitive detections. Cls Loss (Classification Loss) evaluates how accurately the model predicts the object classes within the bounding boxes. Cross-entropy or focal loss is typically used to minimize the difference between predicted and true classes.

## 4 Results

Our seven models were trained on the custom dataset. These models included Faster R-CNN_R_50, Faster R-CNN_R_101, Faster R-CNN_X_101, RetinaNet_R_50, RetinaNet_R_101, Yolov8l, and Yolov8x. To assess the performance of the trained models, 452 images were used as a test dataset. Figures 7, 8 depict the time consumed for models’ training and testing and the losses incurred during the training process.

Furthermore, Figure 9 illustrates the confusion matrices describing the predictive accuracy of the proposed models across eleven classes of in-classroom behaviors, along with relationships between the predictions and their accuracy. This figure illustrates the true labels, predicted categories, and the correct detection rates as diagonal elements. Further different levels of variation in the precision and recall scores of Detectron2 models are presented in Table 3. These metrics reflect the models’ capabilities at various IoU thresholds, reflecting their ability to localize interactions accurately. Additionally, the models’ AP scores across all eleven classes are displayed in Figure 10.

**Table 3:** AP and AR values of the Detectron2 models.

Detectron2 models	AP at IoU = 50–95	AP50	AP75	APs	APm	APl	AR	ARs	ARm	ARl
Faster R-CNN_R_50	48.8	72.2	53	46.4	49.1	53.5	55.9	55.2	55.4	59.6
Faster R-CNN_R_101	49.1	71.398	53.793	48.647	48.796	52.828	56.2	55.8	55.8	58.9
Faster R-CNN_X_101	49.7	72.4	55.3	42.2	49	37.6	55.7	55.1	55.1	39.4
RetinaNet_R_50	48.5	70.5	52.9	48.63	48	55.11	54.4	55.6	53.7	57.6
RetinaNet_R_101	48.65	70.58	53.74	50.47	47.35	55.10	54.6	54.7	53.2	61.3

## 5 Discussion

The results presented in Table 4 and Figures 7–11 illustrate distinct performance patterns among the object detection models, particularly between the YOLOv8 variants and the Detectron2-based models. While YOLOv8x demonstrated superior accuracy and speed, the discussion here focuses not just on these quantitative differences but also on their implications for practical classroom applications and future system improvements. The consistent performance of YOLOv8 models suggests a meaningful advancement in balancing precision with efficiency for real-time detection. The architectural improvements in YOLOv8x—such as enhanced feature maps and streamlined inference—may support broader scalability, especially in school environments with limited computing resources. This positions YOLOv8-based frameworks as promising candidates for near real-time feedback systems in teacher evaluations, a use case often constrained by computational latency.

On the other hand, the more complex architecture of Faster R-CNN led to increased training and inference times Figure 7, which, although tolerable in research settings, may hinder deployment in resource-constrained educational institutions. These observations highlight a trade-off: higher detection precision from multi-stage detectors may not always justify the increased computational demand when scaled to multiple classrooms or extended recording sessions. Performance across categories also provides insight into model strengths and weaknesses. The high accuracy observed in categories such as *No_Book*, *Opened_Book*, and *Teacher_Explains* confirms the models’ effectiveness in recognizing distinct visual patterns. However, the consistent underperformance in detecting *Student_Reads* and *Student_Writes* suggests that the current models struggle with subtle, low-motion, or overlapping interactions. These are precisely the types of interactions that are most pedagogically meaningful, indicating engagement, attention, and comprehension—and thus their omission limits the models’ evaluative depth.

Critically, the failure of Detectron2 models to identify the *Worksheet* class as shown in Figure 10 raises concerns about how class imbalance and visual similarity affect learning dynamics. This reflects a broader issue in educational datasets: nuanced behaviors often occur less frequently, yet they carry disproportionate value in performance assessment. Future work should therefore not only address dataset balancing but also prioritize the modeling of underrepresented behaviors that are pedagogically significant. Comparative analysis with existing literature reinforces these insights. While Bai et al. (2022) reported slightly higher accuracy (90%), their work addressed fewer interaction types. Our model evaluated eleven distinct classroom behaviors, five of which achieved over 90% accuracy—without ensemble methods—suggesting greater behavioral coverage. In contrast, Guo et al. (2021) achieved only 81% accuracy across a narrower range of actions. This indicates that our approach offers broader utility despite small trade-offs in maximum accuracy. These findings align with the emerging consensus that interpretability and context-specific applicability may matter more than marginal performance gains in educational AI applications. Importantly, the training process revealed early signs of overfitting in the Faster R-CNN models, with high training accuracy not translating to equal inference performance. This necessitated additional post-processing strategies such as Non-Maximum Suppression (NMS) and confidence thresholding. The observation underscores a need for models with better generalization, particularly in real-world, non-curated classroom environments where visual noise and occlusions are commonplace.

The limitations observed extend beyond detection scores. Misclassification of visually similar categories (e.g., *Closed_Book* vs. *Worksheet*), as well as confusion between teacher and student actions, reflect a broader challenge: context-blind models struggle to reason about role-based or behaviorally complex interactions. These findings emphasize the need for spatiotemporal modeling and multimodal integration—combining vision with audio or textual data—to disambiguate similar-looking behaviors that differ by context. Looking forward, expanding the dataset to include more diverse classroom layouts, student demographics, and interaction types will be essential. Moreover, incorporating weakly supervised learning could reduce the annotation burden, especially for rare behaviors. Given the promising performance of YOLOv8, we plan to explore its newer variants (e.g., YOLOv9, YOLOv10, YOLOv11) to evaluate potential gains in both speed and interpretability. To support holistic performance evaluation, future work will also explore complementary technologies. These include gesture recognition [e.g., GestureTeach (Liu et al., 2024)], pose estimation, and attention-aware models like MSSTANet (Xiao et al., 2024) and EAPT (Lin et al., 2021), which can capture the temporal progression of interactions and better reflect classroom dynamics. Integrating IoT-based audio and environmental sensors could further enhance model reasoning, creating opportunities for cross-modal validation and more accurate teacher performance analytics.

In addition to detection, our models generate class-wise predictions for eleven distinct behaviors, effectively serving a dual role as a classification layer for teacher-student interactions. While not the final objective of this study, these classification outputs form a critical bridge to a future phase of research currently underway. The upcoming study will analyze category-wise performance to refine the interaction taxonomy—merging semantically similar classes, removing ambiguous ones, and assigning weighted pedagogical importance based on expert input. These refinements aim to support a robust scoring framework that transforms detected behaviors into quantitative indicators of teacher performance. This multi-stage approach will evolve the system from descriptive analytics to prescriptive decision-support, ultimately aligning AI outputs with established educational evaluation standards.

In summary, while this study demonstrates that current object detection models—particularly YOLOv8—can serve as foundational tools for automated classroom analysis, critical limitations remain. The novelty of this work lies not in the models themselves, but in their tailored application to the educational domain, using a uniquely annotated classroom dataset aligned with teacher performance indicators (T-KPIs). Addressing the remaining challenges will require not only model-level innovation but also a rethinking of how complex, subtle, and high-value teacher-student interactions are defined, detected, and interpreted in context.

## 6 Conclusion

Overall, T-KPI analysis plays an important role in improving educational outcomes, accountability, professional growth, and evidence-based decision-making. AI-based systems can provide objective and reliable evaluations, helping to identify areas that need improvement and enhance the quality of education offered to students. CV systems allow computers to interpret and understand visual information from their surroundings like humans do. By tracking movements, gestures, and interactions between teachers and students within a classroom, CV makes it possible to evaluate teacher performance systematically. This study examined three well-known object detection algorithms to assess their ability to detect in-classroom interactions accurately. It was demonstrated that by carefully annotating the dataset, it is possible to correctly detect these interactions.

There were eleven classes (labels) annotated to train the model, with YOLOv8x, a single-shot detector, showing the greatest performance, with a mAP value of 85.8%. RetinaNet_50 and RetinaNet_101, both single-shot detectors, delivered the poorest performances, with mAP values of 70.5 and 70.6%, respectively. Two heavyweight models from the YOLO family were chosen: YOLOv8x and YOLOv8l, which are extremely fast and can effectively detect objects of different scales. For Detectron2, we applied two algorithms, Faster R-CNN and RetinaNet, and adopted five models from these algorithms. For further improvement of the YOLOv8 models, it is recommended to include images from all classes, particularly from classes with smaller sample sizes.

## Data Availability

The datasets presented in this article are not readily available due to strict privacy restrictions, as outlined in the consent form signed by participants to ensure their confidentiality and data security. Requests to access the datasets should be directed to arwa.s.almubarak@gmail.com.
